# Filling Teeth

**Published:** 1871-05

**Authors:** 


### Editorial.

### FILLING TEETH.

The process of filling teeth, requires for its efficient accomplishment great skill and care, very few who attempt to perform this operation, have a complete apprehension of all that is involved in it. The nature and peculiarities of the tissue operated upon, should be taken into account, together with all the favorable and unfavorable influences or circumstances that may be presented.

It is proposed in these lines to present two or three points in this operation, that are not generally observed, and the first is, that of filling teeth, especially the anterior, under glasses of a high magnifying power, or from two to three diameters.

We have for several years, used glasses for definition, but without any material magnifying power, and operated as well as we ever did, with the best unaided vision.

We have recently, however, been using glasses that magnify about two diameters, and find it very much easier to operate, both in the formation of cavities, and in the filling. There is far more precision in the manipulation, and more perfect results are obtained throughout. So great is the difference, that the cavities which were before annoying and very difficult to fill, are now filled with pleasure, and with a degree of certainty as to results, that before were very seldom experienced; any one has but to test the method to appreciate its value.

In our experiments thus far, we have combined two ordinary sets of glasses, but a better appliance could doubtless be made, by arranging two or three lenses in one frame. This principle is as applicable and as useful to those who have not lost difinition by age or otherwise, as to those who have. We may refer to this subject again.

Another point in the operation of filling teeth, well worthy of consideration, is the ease and facility, with which all borders and margins of cavities may be protected by the use of heavy foils. In almost every instance, where the enamel has been cut away, and the dentine exposed about the orifice of the cavity in the preparation for filling, it should be completely covered by a heavy layer of gold. This can in all cases be done, by filling the cavity with gold in any form it may be desired, even with its borders, and then applying Nos. 60 to 80 gold foil, in plain pieces, single thickness, a little larger than the surface to be covered. Weld this thoroughly to the surface of the gold already introduced, then fold over the edge a little all round, and work it down solidly on to the edge of dentine and enamel. Apply successive layers of the foil in this manner, until a sufficient fullness is given to the entire surface of the filling, and a strong flange formed on the edge of the wall. Foot instruments of various forms will be required, for consolidating these single layers of gold, especially in proximal fillings. Without the right instruments, it will be useless to attempt this method.

It will oftentimes occur, in building up this surface portion, that unevenness of surface will occur, in spite of the utmost care. In order to remedy this, a thin separating file may be used, dressing the surface truly, then apply two or three additional pieces, consolidating all with the serrated condenser, except the last piece, which should be condensed with a foot burnisher. If care has been exercised, the file will scarcely be needed at all on the surface of such a filling, except for finishing round the borders, after which, with the stone, corrundum tape, and the proper polishers, a most perfect finish can be produced.

The chief point in this method is the protection that is afforded to the margin of the cavity. How often is it, that operations faultless in all other respects, ultimately fail because thin or feeble edges about them give way, but a little it may be, yet sufficient to give lodgment to foreign substance, and a beginning to decay; whilst had they been protected as above suggested, no such accident could have occurred.

In very many cases of filling proximal cavities of both posterior and anterior teeth, it is found difficult after the entire filling is introduced, to work over the entire surface of the filling, in such a manner as to give it a complete finish, without making more space between the teeth than is desirable, especially is this true, of that part of the filling, toward and at the cervical border. To overcome the difficulty of making a perfect finish, that so frequently occurs here, after a fourth to a third of the filling is introduced, which in all cases is upon the cervical wall, a complete finish of its proximal surface should be made, the entrance and approach to it for this purpose, is far more easy than after the filling is wholly introduced. T.

